# Delayed Presentation of Post-traumatic Multiorgan Left Diaphragmatic Hernia: A Case Report and Literature Review

**DOI:** 10.7759/cureus.26814

**Published:** 2022-07-13

**Authors:** Mohd Yunus Shah, Ahmed A Abdrabou, Prakash Obalappa

**Affiliations:** 1 Department of General Surgery, Minimal Invasive and Bariatric Surgery, Al-Ahli Hospital, Doha, QAT; 2 Department of ENT and Head Neck Surgery, Al-Ahli Hospital, Doha, QAT

**Keywords:** polytrauma, blunt trauma abdomen, abdominal trauma, delayed diaphragmatic rupture, traumatic diaphragmatic injury, diaphragmatic hernia

## Abstract

Post-traumatic diaphragmatic injuries can present as diaphragmatic hernia with herniation of abdominal viscera into the thoracic cavity. It is challenging for trauma surgeons to identify the delayed presentation of post-traumatic diaphragmatic injuries which require a high index of suspicion in patients who are at risk. We report a rare case of delayed diagnosis and management of post-traumatic diaphragmatic hernia in a polytrauma patient with a concise review of the literature. The patient presented after two years of post-traumatic thoracoabdominal injury due to a road traffic accident with breathing difficulty. On investigations, it was a large diaphragmatic hernia with herniation of abdominal contents into the left thoracic cavity. Laparotomy was performed with a reduction of abdominal contents from the left side of the chest along with mesh repair of the large diaphragmatic hernia. Postoperatively, the patient recovered well. The literature suggests that there should be a high level of suspicion of diaphragmatic injuries, especially when dealing with thoracoabdominal trauma or polytrauma patients. Post-traumatic diaphragmatic injuries, though rare, can lead to high morbidity or mortality if not treated on time.

## Introduction

Post-traumatic diaphragmatic injuries can present with symptoms of diaphragmatic hernia due to the herniation of abdominal viscera into the thoracic cavity. Identifying the delayed presentation of post-traumatic diaphragmatic injuries is challenging for trauma surgeons because it requires a meticulous examination of at-risk patients. A diaphragmatic hernia has been reported in 1-7% of patients with blunt abdominal trauma and 10-15% of cases involving penetrating abdominal injuries [1]. While post-traumatic diaphragmatic hernias are rare, they can lead to high morbidity or mortality if not treated promptly.

Diaphragmatic injuries should be suspected when dealing with thoracoabdominal trauma or polytrauma patients. Traumatic diaphragmatic injuries can be missed initially due to other associated injuries. The presentation of diaphragmatic hernia is commonly delayed compared with other organ injuries when the abdominal organ herniates into the pleural cavity. Traumatic diaphragmatic hernia is rare, difficult to diagnose, and can lead to delay in the diagnosis resulting in high morbidity and mortality rates [2].

Here, we report the case of a 57-year-old male patient with delayed presentation of post-traumatic diaphragmatic hernia on the left side who presented two years after polytrauma. The case was examined in detail concerning presentation, investigation, and management. In addition, previous case studies of late presentation of diaphragmatic injuries were also reviewed. The patient had a large diaphragmatic hernia with multiorgan herniation (small bowel loops, spleen, colon, tail of the pancreas) into the left thoracic cavity leading to a collapse of the left lung. Surgical repair of the diaphragmatic hernia was done after repositioning of all the herniated organs into the peritoneal cavity.

## Case presentation

A 57-year-old male was admitted with complaints of difficulty in breathing, hiccups, and cough for 15 days increasing in intensity. The patient had suffered thoracoabdominal trauma due to a road traffic accident two years ago for which he was admitted to the intensive care unit (ICU) and investigated and managed conservatively. A computed tomography (CT) of the scan abdomen did not reveal any intraabdominal injury at the time. The patient recovered well and was discharged.

**Figure 1:**
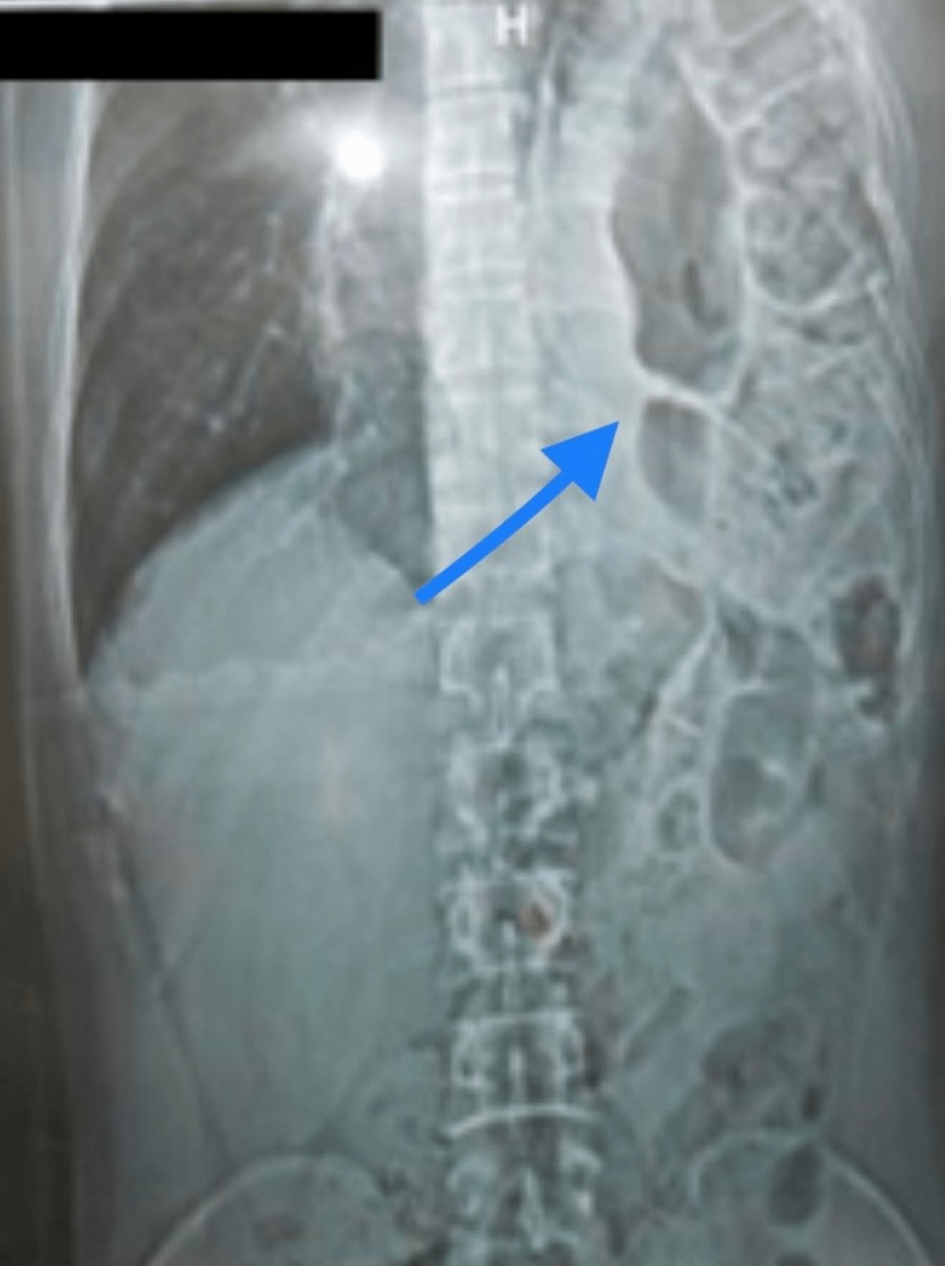
X-ray of the chest and abdomen showing herniation of bowel loops into the left side of the chest with the collapsed left lung.

On examination, the patient was restless and was experiencing difficulty in breathing. His heart rate was 96 beats per minute, respiratory rate was 28 breaths per minute, and on-air oxygen saturation was 70-80%. On chest auscultation, gurgling sounds were heard on the left side, and systemic examination was normal. An X-ray of the chest and abdomen revealed the presence of bowel loops in the left thoracic cavity, while the left dome of the diaphragm was not visualized. The left lung had collapsed (Figure 1).

**Figure 2:**
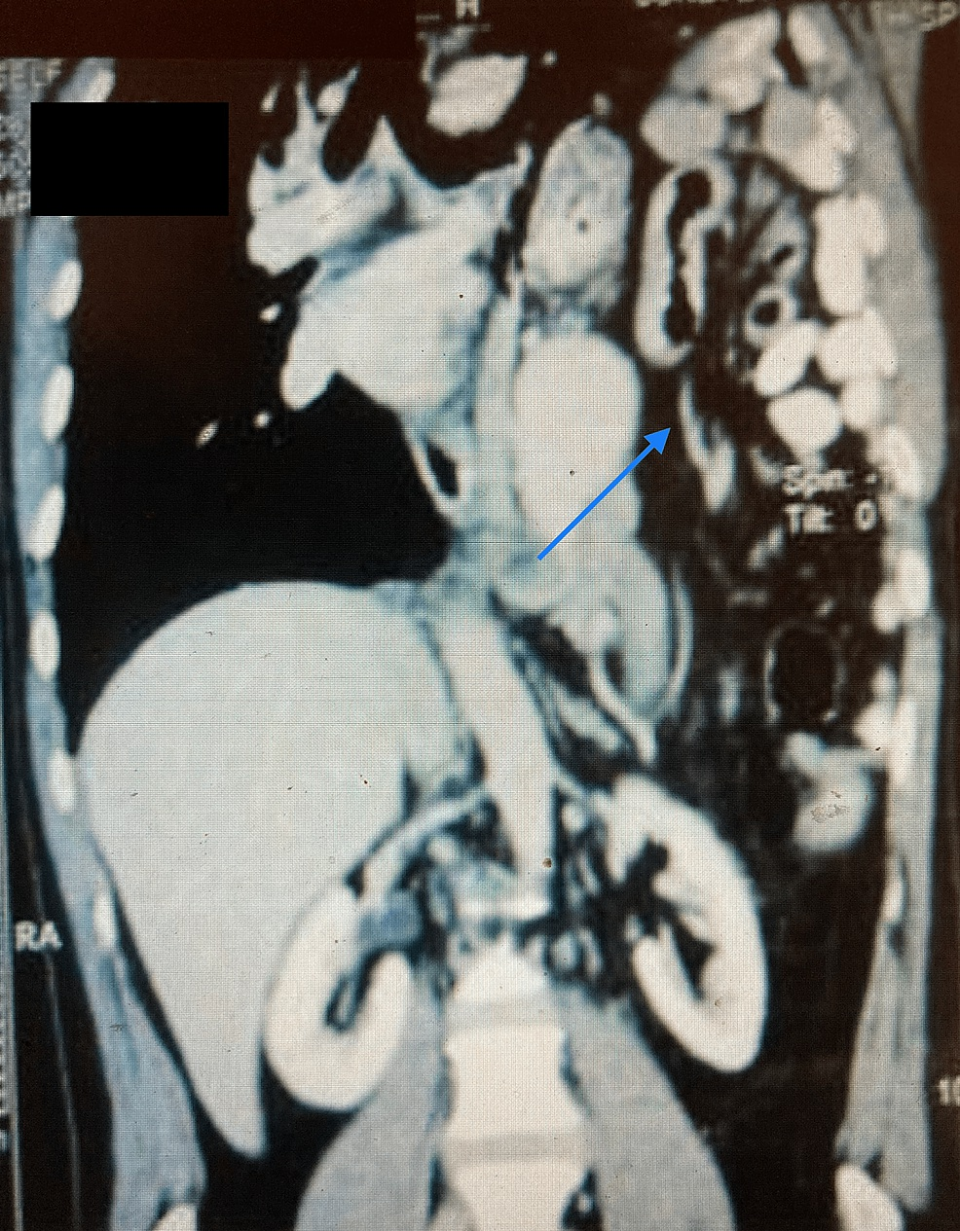
Contrast-enhanced computed tomography of the abdomen and chest showing a large diaphragmatic hernia with herniated bowel loops in the transverse colon, the splenic flexure of the colon with mesentery, the spleen, and the tail of the pancreas, as well as the collapsed left lung.

The thoracic ultrasound imaging suggested loculated pleural effusion on the left side with the consolidation of the lung in the mid-lower zone, with a fluid volume of 244 cc and multiple pockets. The contrast-enhanced CT scan of the abdomen and thorax revealed a large diaphragmatic hernia with herniation of the small bowel loops, the stomach, part of the transverse colon, the splenic flexure of the colon, the spleen, and the tail of the pancreas into the left side of the thoracic cavity, as well as the collapsed left lung (Figure 2).

**Figure 3:**
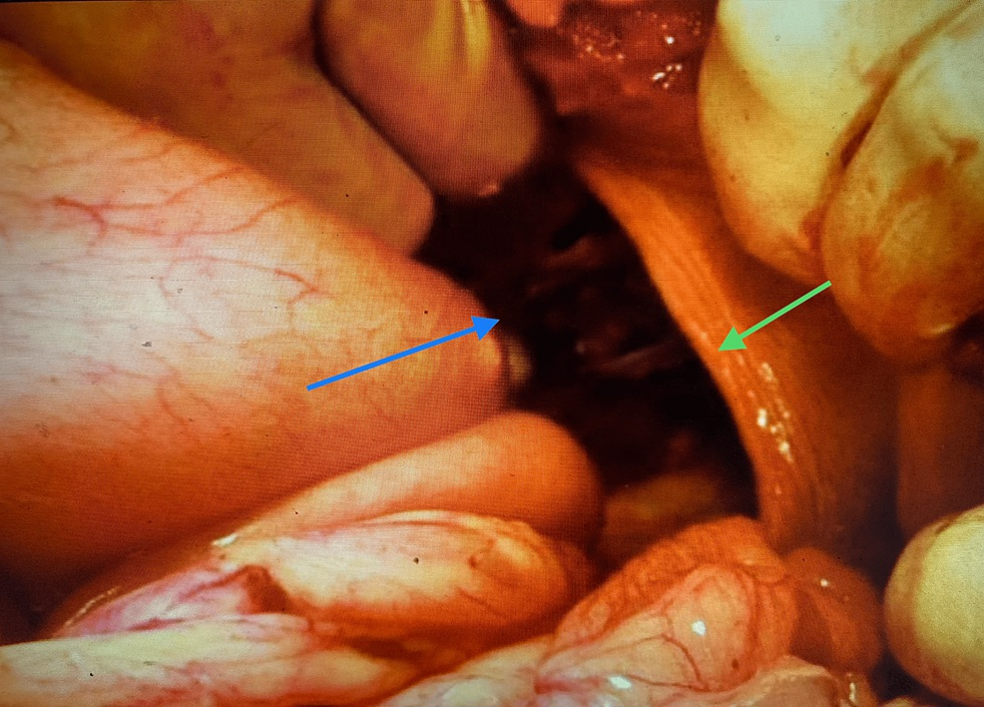
Large diaphragmatic hernia (green arrow) with herniation of the small bowel loops, colon, spleen, and tail of the pancreas into the left side of the thoracic cavity (blue arrow).

The patient was prepared for surgery after obtaining informed consent and an anesthetic evaluation. Under general anesthesia, a left subcostal incision was made. Intraoperatively, a large diaphragmatic hernia was present with herniation of the abdominal organs, two-thirds of the stomach, the small bowel, part of the transverse colon, the splenic flexure of the colon, the spleen, and the tail of the pancreas into the left pleural cavity through a diaphragmatic defect of 10 × 8 cm (Figure 3).

**Figure 4:**
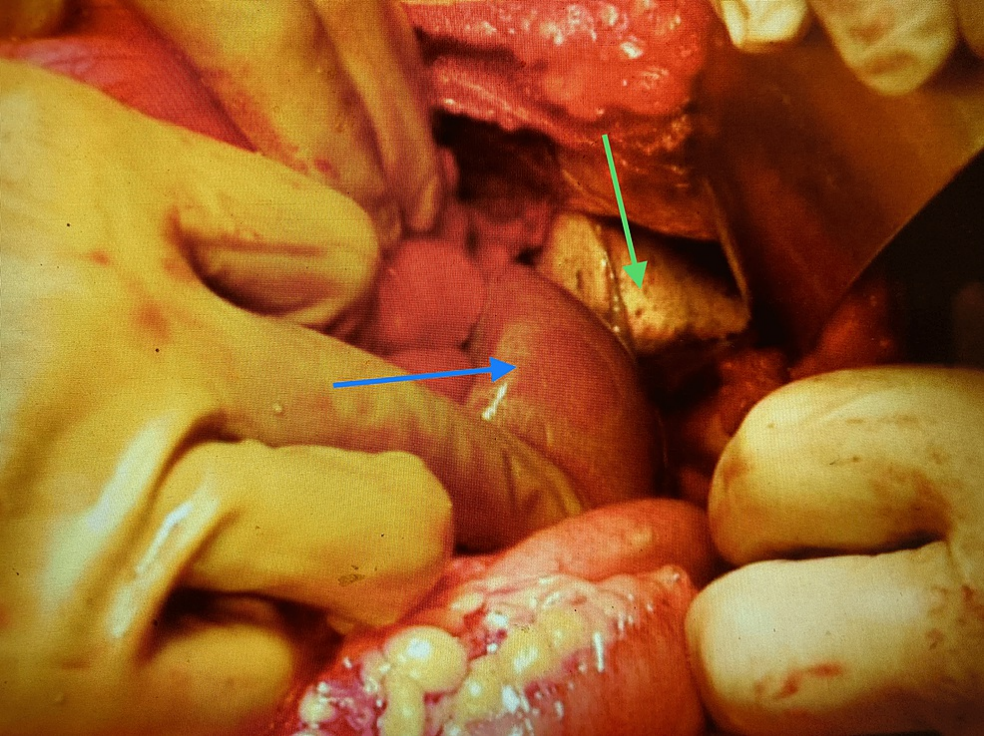
Left diaphragmatic herniation of bowel loops, colon, and spleen (blue arrow) with the collapsed left lung (green arrow).

The collapsed left lung along with the herniated bowel loops and the spleen could be seen through the large diaphragmatic hernia (Figure 4).

**Figure 5:**
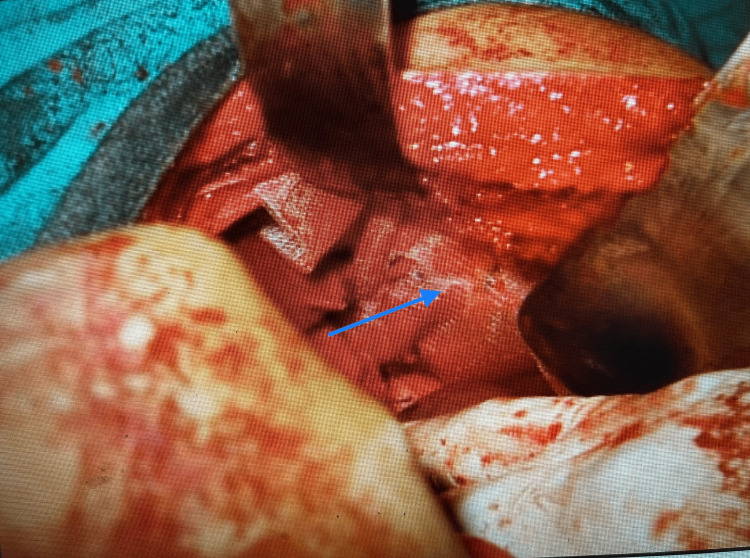
Left diaphragmatic hernia repair with mesh following adhesiolysis and the reduction of the contents into the peritoneal cavity.

All the contents were reduced into the peritoneal cavity after checking the vascularity, which was found to be normal. The entire bowel was recovered into the abdominal cavity after performing adhesiolysis. The defect in the diaphragm was sutured without tension using polyglactin 2-0 and was reinforced with 15 × 15-cm mesh sutured all around the defect (Figure 5).

An intercostal drain was placed in the left pleural cavity. Following the surgery, the patient was managed in the ICU, with oxygen supplementation, chest physiotherapy, and breathing exercises. The patient recovered well. The postoperative chest X-rays were normal, and the patient was discharged on the fifth postoperative day, without difficulty in breathing or any other symptoms.

## Discussion

Traumatic rupture of the diaphragm is rare, critical, and often difficult to diagnose [3]. Trauma due to road traffic accidents remains the main cause of these traumatic diaphragmatic ruptures (TDRs). Even though there are various indirect radiological signs to diagnose TDR, the diagnosis is missed in up to 60% of the cases during the initial imaging [4,5]. Among the blunt trauma patients, 0.8% to 1.6% have traumatic rupture of the diaphragm [6].

The majority of the TDRs (69%) are seen on the left side, with 24% and 15% occurring on the right side or bilaterally, respectively [7]. In an emergency, there is a possibility of missing the diagnosis of TDR due to other associated severe injuries such as head injury, hemothorax/pneumothorax, or head-neck trauma. If the TDR is missed it leads to diaphragmatic hernia. The delayed presentation of post-traumatic diaphragmatic hernia can vary from months to years after the initial injury with the herniation of abdominal viscera into the thoracic cavity, obstruction, incarceration, and perforation leading to respiratory distress [8]. In our case, the patient presented after two years of the initial injury.

The exploratory laparotomy approach is the gold standard approach in emergency settings for the repair of diaphragmatic injuries associated with visceral injuries [9]. In delayed, long-standing cases of TDR, thoracotomy is preferred as it is easier to release the adhesions formed by the herniated bowels with the intrathoracic viscera through the thoracotomy approach [10]. The abdominal approach is preferred by some surgeons as the adhesiolysis or resection anastomosis is easier in this approach. The choice of approach depends upon the surgeon’s preference [11]. In our case, the abdominal approach was preferred as there were adhesions present in the abdominal part of the herniated colon and the small bowel.

## Conclusions

Post-traumatic diaphragmatic hernias can be misdiagnosed and can remain asymptomatic for months to years. In the case of thoracoabdominal injuries, a diagnosis of traumatic rupture of the diaphragm should be carefully considered because it can be masked by other severe injuries. A high index of suspicion should be kept for diagnosing traumatic diaphragmatic injuries. The CT scan of the chest and abdomen is the preferred imaging investigation for the diagnosis, while the surgical treatment includes the reduction of any visceral hernia, repair of the diaphragm, and restoration of the circulation, breathing, and digestive functions. The type of surgical approach adopted, whether thoracotomy, laparotomy, or minimal access surgery, will depend upon other associated injuries, as well as the surgeon’s preference or expertise.
